# Supplementary material for: Efficacy of superimposing neuromuscular electrical stimulation onto core stability exercise in patients with nonspecific low back pain: A study protocol for a randomized controlled trial
Source: PLoS One. 2025 May 7;20(5):e0322398. doi: 10.1371/journal.pone.0322398 (PMC12057853; doi:10.1371/journal.pone.0322398)
Supplement: S2 File — (DOCX) [file pone.0322398.s002.docx]

研

究

方

案

研究名称：神经肌肉电刺激叠加训练对非特异性腰痛患者核心肌群延迟激活的作用及机制研究

研究单位：浙江大学医学院附属邵逸夫医院

研究科室：康复医学科

主要研究者：李永忠

方案版本号：V1.0

方案版本日期：2024年04月06日

**一、研究背景**

下腰痛（Low Back Pain，LBP）作为全球范围内导致残疾的普遍原因，对医疗保健系统构成了挑战，同时也是一个重要社会问题。据估计，75%-85%的人在一生中至少经历过一次腰痛。截至到2017年，全球LBP患病率约为7.5%，相当于约5.77亿人患有LBP。在排除影响腰椎的特定疾病等病因后，多达90%的LBP患者无法做出特异性诊断，因此这类患者被归类为“慢性非特异性下腰痛”（chronic on specific low back pain，CNLBP）。CNLBP患者因活动和工作能力的丧失而导致残疾，不仅成为一个主要的个人健康问题，而且由于暂时的经济影响或永久性的工作能力丧失，将带来巨大的经济损失和沉重的生活负担。

在过去的30年之中，考虑到尚无确凿证据表明以疼痛为中心的临床干预措施能够显著减轻CNLBP患者的腰背痛负担，下腰痛的临床干预重心已经从疼痛治疗转向通过改善活动和参与来提高功能。目前CNLBP的治疗循证指南强调坚持运动，具体措施包括肌力增强和耐力提高、特定躯干肌肉激活、运动控制、有氧训练或多模式运动等。因此，通过运动训练干预以纠正异常的活动和参与，从而达到缓解疼痛和改善功能的目的，已经成为治疗CNLBP患者最为重要的手段之一。尽管现有的运动训练方式在短期内表现出显著疗效，但是其长期效果难以维持，而且每次治疗所需的时间较多，这使得大多数患者难以长期保持坚持。

核心肌群的延迟激活是CNLBP患者在运动适应后引起姿势控制障碍的主要因素之一。在CNLBP患者中，长期疼痛引起的运动适应会改变协同肌肉内部或之间的活动分布，影响本体感觉功能，改变运动皮质的兴奋性及运动反应计划。这些变化可能导致组织负荷的增加，引起组织刺激和结构变化，随着时间推移，进一步导致姿势控制障碍。延迟激活不仅会损害脊柱稳定系统，导致脊柱组织应变的增加，还会加强躯干肌肉协同收缩，从而引发肌肉疲劳，导致腰椎的不稳定。这种腰椎不稳性使得个体在长时间的错位姿势或腰椎反复运动时剪切力增加，增加韧带组织的损伤风险，提高患者再次受伤的可能性。CNLBP患者姿势控制障碍不仅在日常站立、坐姿和行走中显著表现，而且在执行具有挑战性的任务时（如单腿站立和功能性活动），也呈现核心肌群激活的明显延迟和代偿性姿势反应。

神经肌肉电刺激（Neuromuscular Electrical Stimulation，NMES）是一种潜在的用于改善核心肌群延迟激活的干预措施，已成功应用于改善损伤个体的脊柱排列、坐姿、躯干稳定性和移动能力。最近研究指出，NMES的运动生理学和临床益处可能是通过感觉运动整合机制实现的。NMES诱发的运动产生的本体感觉信号的增加，激活感觉运动网络，从而提高皮质脊髓兴奋性，促进相关神经网络更大范围的激活，进而导致包括肌肉延迟激活和力量在内的功能改善。

神经肌肉电刺激叠加训练（NMES+训练）是在电刺激治疗基础上结合个体的自主收缩，构成一种潜在的互补训练模式。已经证明，与仅使用NMES或仅进行随意运动训练相比，NMES+训练在改善运动表现方面更有效。此外，在时间花费方面，NMES+训练表现出显著的积极效果，并能够带来额外的神经和生理效应。在NMES+训练期间，脊髓兴奋性的增加与增强的下行驱动相结合，可以反映出NMES+训练对肌肉收缩能力和运动单位募集的积极影响。所以，NMES+训练是一种改善自主收缩、提高肌肉功能和增强运动控制的良好选择，有望在CNLBP患者康复方面取得显著的临床成果。

以往关于NMES+训练效应的研究通常集中在运动人群和骨科患者的肌肉激活程度或者募集形式上，对于CNLBP患者，研究主要关注于缓解疼痛、改善功能等方面的行为或现象。然而，至今为止尚未见NMES+训练对于CNLBP患者核心肌群肌肉募集形式（尤其是延迟激活效应）的相关报道。鉴于NMES+训练对肌肉功能改善的前期相关研究报道，本项目的主要目的在于观察NMES结合核心训练对CNLBP患者核心肌群延迟激活的影响，并且探讨相关的作用机制，为发展更有效和更加全面的CNLBP患者治疗方案提供新思路、新的技术平台。

**二、研究目的**

本项目主要目的在于观察NMES结合核心训练对CNLBP患者核心肌群延迟激活的影响，并探讨相关的作用机制，为发展更有效的和更加全面的CNLBP患者治疗方案提供新思路、新的技术平台。

**三、研究设计与方法**

**3.1 研究对象**

本研究拟纳入于浙江大学医学院附属邵逸夫医院康复医学科就诊且诊断为CNLBP患者，相关纳入排除标准如下。

**3.1.1 纳入标准**

（1）年龄在18 - 60周岁。

（2）符合2016版中国康复医学会脊柱脊髓专业委员会专家组《中国急/慢性非特异性腰背痛诊疗专家共识》中有关CNLBP的诊疗标准。

（3）视觉模拟评分（VAS）得分≤6分。

（4）近1个月未接受过本研究所采用的治疗干预措施。

（5）不经常服用镇痛药（4天/周）。

（6）没有影响肌肉代谢的药物治疗或疾病（例如糖皮质激素）。

（7）听力及心理正常，且配合依从性高。

（8）自愿参加本研究，并签署知情同意书。

**3.1.2 排除标准**

（1）弱势群体，包括精神疾病者、认知损伤者、危重患者、未成年人、孕妇、文盲等。

（2）合并心脑血管、肝、肾等严重基础疾病。

（3）合并骨关节炎疾病、痛风、肿瘤、急性创伤或骨折等影响日常生活能力的参与者。

（4）既往脊柱手术史。

（5）NMES禁忌证（起搏器、水肿、感觉异常、血栓栓塞等）。

（6）正在参加其它CNLBP患者干预临床实验的参与者。

**3.1.3 退出标准**

（1）受试者自行退出本研究。

（2）研究者认为受试者不适合继续参加本研究。

**3.2 研究内容**

**3.2.1 受试者分组**

本研究将受试者随机分为同步干预组和假刺激干预组两组。

同步干预组：神经肌肉电刺激治疗与核心力量训练同时进行。

假刺激干预组：假神经肌肉电刺激治疗与核心力量训练同时进行。

**3.2.2 干预过程**

**3.2.2.1 核心力量训练**

核心训练内容包括在仰卧位、俯卧位和四足跪位的腹横肌激活训练、桥式和平板支撑等（详见表1）。每次进行8种不同的训练动作，每个动作维持6秒，然后休息6秒，共持续运动20分钟。每个动作进行1组，10个/组。每周治疗3 天，共治疗18次，为期6周。

**3.2.2.2 神经肌肉电刺激治疗**

使用神经肌肉刺激器（EN-Stim 4；ENRAF-NONIUS B.V.公司，布鲁森, 荷兰）对躯干双侧多裂肌和腹横肌/腹内斜肌实施电刺激。腹部电极的一处位于沿腋中线髂嵴上1cm处，另一处位于髂前上棘上方和内侧2cm处；腰部电极位于L4以及L5棘突间旁开约2cm处。电极片规格为5cm × 5cm水凝胶表面电极。刺激波宽为200微妙，频率50Hz，整体收缩-放松周期为上升1s，收缩4s，下降1s，静息6s，共持续20分钟。电流强度以能引起最大程度的肌肉收缩而不产生不适，如烧灼感或严重的强直性疼痛为宜。核心训练在收缩期进行，静息期停止。每周治疗3天，共治疗18次，为期6周。

假神经肌肉电刺激：电流强度被设定在一个不引起任何肌肉收缩的最低水平。由于患者没有感觉到收缩，因此治疗师在设备指示灯上观察到的阶段转换期间，指导患者开始和停止运动。每周治疗3天，共治疗18次，为期6周。

**表1 核心训练具体内容**

| **第1-2周** | **第3-4周** | **第5-6周** |
| --- | --- | --- |
| 仰卧位腹横肌束带训练：肚脐朝向背部运动 | 仰卧位腹横肌束带运动下伴对侧上下肢抬起（不接触地面） | 仰卧位腹横肌束带运动下双桥 |
| 仰卧位腹横肌束带运动下伴脚后跟滑动 | 仰卧位腹横肌束带运动下双桥 | 仰卧位腹横肌束带运动下伴空中自行车 |
| 仰卧位腹横肌束带运动下伴下肢抬起 | 仰卧位腹横肌绷紧伴单桥（左腿） | 仰卧位腹横肌束带运动下伴卷腹（手触膝） |
| 仰卧位腹横肌束带运动下伴对侧上下肢抬起 | 仰卧位腹横肌绷紧伴单桥（右腿） | 手膝跪位腹横肌绷紧伴同时抬对侧上下肢 |
| 仰卧位腹横肌束带运动下伴双桥 | 屈膝下侧桥（左侧） | 膝伸直下侧桥（左侧） |
| 手膝跪位腹横肌束带运动下伴抬上肢 | 屈膝下侧桥（右侧） | 膝伸直下侧桥（右侧） |
| 手膝跪位腹横肌束带运动下伴抬下肢 | 俯卧位腹横肌束带运动下伴对侧上下肢抬起 | 俯卧位腹横肌束带运动下伴对侧上下肢抬起 |
| 手膝跪位腹横肌束带运动下伴对侧上下肢同时抬起 | 手膝跪位腹横肌束带运动下伴对侧上下肢同时抬起 | 平板支撑 |

**3.2.3 观察指标**

本研究涉及的检查评估项目均为临床常规检查评估项目，具体如下。

**3.2.3.1 主要观察指标**

表面肌电图评估（sEMG）：于治疗前及治疗6周后采用芬兰产MegaWin ME6000-T8型表面肌电系统进行肌电检测，观察每位受试者在抬物动作过程中核心肌群激活程度以及起始激活时间的改变。

**3.2.3.2 次要观察指标**

在开始治疗前、治疗6周后对每位受试者进行如下评估：1）使用肌骨超声（SONIMAGE HS1，KONICA MINOLTA，上海，中国）评估多裂肌和腹部肌肉厚度以及多裂肌横截面积；2）使用本地感觉测试评估腰背部本体感觉改善情况；3）使用Owestry功能障碍指数（ODI）问卷表评估腰椎功能；4）使用视觉模拟评分（VAS）量表评估疼痛情况。

在治疗后6个月进行随访评估。

**3.2.4 统计学分析**

采用SPSS 24.0 for windows软件进行数据处理。计量资料采用均数±标准差进行统计学描述。采用单因素方差分析或卡方检验分析三组基线指标的差异情况。采用双因素方差分析对不同组别的评价指标进行组间比较。应用皮尔逊双变量相关性分析评估核心肌群延迟激活变化与相关评估结果之间的关系。所有统计检验均采用双侧检验，P＜0.05为差异有统计学意义。

**四、样本量计算**

本研究的样本量根据Songjaroen等报道的研究结果确定。神经肌肉电刺激联合运动控制训练可以改善复发性下腰痛患者的腰椎多裂肌激活。计算基于LM激活改善的预期效应量，具有较大的效应量（Cohen's dz = 0.7），统计功效为80%，显著性水平为5%，标准差为0.05点。使用G*Power 3.1软件进行分析，计算出至少需要43名参与者。考虑到20%的预期脱落率，本研究将招募52名参与者（试验组26名，对照组26名）。

**五、数据管理和保密**

有关受试者身份相关的所有信息资料均予以保密，相关资料在相关法律和/或法规允许的范围之外不对外公开。

**六、知情同意**

每位受试者在入选本研究之前，负责谈知情同意的研究者要以书面文字形式向其完整、全面地介绍本研究的目的、性质、程序以及可能的受益及风险等，应让受试者知晓他们有权随时退出研究。入选前，每位受试者应被充分知情，且有充分的时间考虑是否参加。受试者自愿参加并签署知情同意书之后方可入选本研究。

**七、不良事件及相关处理措施**

本研究使用的神经肌肉电刺激治疗属于低频电疗法的一种，研究者在对受试者进行电疗过程中将严格按照仪器设备说明书操作要求进行相关的电极片放置以及电刺激方案的调整，极少数受试者可能会有一些非常小的风险，如电灼伤等。若受试者发生皮肤被灼伤，本研究团队会为受试者提供皮肤电灼伤的相关处理，包括使用无菌纱布进行包扎清创和使用碘伏等对患处进行消毒处理等。

本研究的核心训练为一种常规针对慢性下腰痛患者的运动训练方式，研究者在对受试者开展核心训练过程中将严格按照运动处方剂量执行，并对训练环境进行规范管理，开展预防跌倒宣教。极少数的受试者可能会出现一些非常小的风险，如肌肉酸痛等。如果受试者发生训练后肌肉酸痛，本研究团队将会为受试者提供相关处理，如推拿按摩、热敷等对酸痛部位进行处理。
