# Supplementary material for: Efficacy of superimposing neuromuscular electrical stimulation onto core stability exercise in patients with nonspecific low back pain: A study protocol for a randomized controlled trial
Source: PLoS One. 2025 May 7;20(5):e0322398. doi: 10.1371/journal.pone.0322398 (PMC12057853; doi:10.1371/journal.pone.0322398)
Supplement: S3 File — (DOCX) [file pone.0322398.s003.docx]

****Study PROTOCOL****

****Study Title:**** Efficacy of Superimposing Neuromuscular Electrical Stimulation onto Core Stability Exercise in Patients with Nonspecific Low Back Pain

****Research Institution:**** Sir Run Run Shaw Hospital, Zhejiang University School of Medicine
****Department:**** Department of Rehabilitation Medicine
****Principal Investigator:**** Yongzhong Li
****Protocol Version Number:**** V1.0
****Protocol Version Date:**** April 6, 2024

### **Research Background**

Low back pain (LBP) is a leading cause of disability worldwide, posing significant challenges to healthcare systems and representing a critical societal issue. It is estimated that 75%–85% of individuals experience LBP at least once in their lifetime. As of 2017, the global prevalence of LBP was approximately 7.5%, equating to 577 million people affected. After excluding specific diseases affecting the lumbar spine, up to 90% of LBP cases cannot be attributed to a definitive diagnosis and are categorized as "chronic nonspecific low back pain" (CNLBP). CNLBP leads to disability due to loss of activity and work capacity, making it not only a major individual health concern but also a significant economic burden due to temporary economic impacts or permanent work incapacity.

Over the past three decades, the focus of clinical interventions for LBP has shifted from pain relief to improving function through enhanced activity and participation, as evidence suggests that pain-centric treatments offer limited benefits. Current evidence-based guidelines emphasize the importance of exercise, including strengthening, endurance training, targeted activation of trunk muscles, motor control exercises, aerobic training, and multimodal exercise programs. Exercise interventions aim to correct abnormal activity and participation patterns, alleviating pain and improving function, making exercise a cornerstone in CNLBP management. While existing exercise regimens show short-term efficacy, their long-term effects are difficult to maintain, and time-intensive therapy sessions often lead to poor adherence.

Delayed activation of core muscles is a key factor contributing to postural control impairments in CNLBP patients. Chronic pain-induced motor adaptations alter muscle coordination, proprioception, cortical excitability, and motor planning. These changes increase tissue load, stimulate structural changes, and exacerbate postural control issues. Delayed activation not only impairs spinal stability but also promotes compensatory muscle co-contractions, leading to fatigue and lumbar instability. This instability increases shear forces during prolonged misalignment or repetitive lumbar movements, raising the risk of ligament injury and re-injury. CNLBP patients exhibit significant postural control deficits during everyday activities (e.g., standing, sitting, walking) and during challenging tasks (e.g., single-leg stance, functional activities), characterized by delayed core muscle activation and compensatory postural responses.

Neuromuscular electrical stimulation (NMES) is a promising intervention for improving delayed core muscle activation. NMES has demonstrated success in enhancing spinal alignment, posture, trunk stability, and mobility in injured individuals. Recent research suggests that the physiological and clinical benefits of NMES may result from sensorimotor integration mechanisms. By increasing proprioceptive signals induced by NMES, sensory-motor networks are activated, enhancing corticospinal excitability and leading to improved muscle activation and function.

NMES combined with voluntary training (NMES+Training) represents a complementary training model that combines NMES with individual voluntary contractions. Studies have shown that NMES+Training is more effective in improving motor performance than NMES or voluntary training alone. It also saves time and provides additional neurological and physiological benefits. During NMES+Training, increased spinal excitability and enhanced descending drive reflect its positive effects on muscle contraction and motor unit recruitment, making it a promising strategy for CNLBP rehabilitation.

While previous studies on NMES+Training have focused on muscle activation or recruitment patterns in athletic or orthopedic populations, CNLBP research has primarily examined pain relief and functional improvement. However, no studies have specifically addressed the effects of NMES+Training on delayed core muscle activation in CNLBP patients. Based on preliminary evidence of its efficacy, this study aims to evaluate the impact of NMES combined with core training on delayed core muscle activation in CNLBP patients, explore its mechanisms, and provide innovative insights and technical platforms for developing more effective and comprehensive CNLBP treatment protocols.

### **Research Objectives**

The primary objective of this project is to investigate the effects of neuromuscular electrical stimulation (NMES) combined with core training on delayed core muscle activation in patients with chronic nonspecific low back pain (CNLBP). Additionally, the study aims to explore the underlying mechanisms of these effects to provide innovative insights and a new technical platform for developing more effective and comprehensive treatment strategies for CNLBP patients.

### **Research Design and Methods**

#### **Study Participants**

This study intends to recruit patients diagnosed with chronic nonspecific low back pain (CNLBP) who are receiving treatment at the Department of Rehabilitation Medicine, Sir Run Run Shaw Hospital, Zhejiang University School of Medicine. The inclusion and exclusion criteria are as follows:

**Inclusion Criteria**

1. Age between 18 and 60 years.
2. Meets the diagnostic criteria for CNLBP as outlined in the 2016 Expert Consensus on the Diagnosis and Treatment of Acute/Chronic Nonspecific Low Back Pain by the Spine and Spinal Cord Committee of the Chinese Association of Rehabilitation Medicine.
3. Visual Analog Scale (VAS) score ≤ 6.
4. No prior exposure to the interventions used in this study within the past month.
5. Does not frequently use analgesics (≤4 days per week).
6. No medication or disease affecting muscle metabolism (e.g., corticosteroid use).
7. Normal hearing and psychological state, with high compliance and cooperativeness.
8. Voluntary participation in this study with signed informed consent.

#### **Exclusion Criteria**

1. Belonging to vulnerable groups, including individuals with mental disorders, cognitive impairment, critical illness, minors, pregnant women, or illiterate individuals.
2. Severe underlying diseases affecting the cardiovascular, cerebrovascular, liver, or kidneys.
3. Presence of osteoarthritis, gout, tumors, acute trauma, or fractures that impair daily activities.
4. History of spinal surgery.
5. Contraindications to NMES, such as pacemaker use, edema, sensory disturbances, or thromboembolism.
6. Current participation in other clinical trials for CNLBP interventions.

#### **Withdrawal Criteria**

1. Voluntary withdrawal from the study by the participant.
2. Determination by the researchers that the participant is no longer suitable to continue the study.

#### **Research Content**

##### Participant Grouping

Participants in this study will be randomly assigned to two groups: Intervention Group, and the Sham Stimulation Group.

****Intervention Group****

Participants will undergo NMES therapy and core strength training simultaneously.

****Sham Stimulation Group****

Participants will receive sham NMES therapy alongside core strength training.

### **Intervention Procedure**

#### ****Core Strength Training****

Core training exercises will include transversus abdominis activation exercises in supine, prone, and quadruped positions, as well as bridging and planking exercises (details in Table 1).

**Protocol:**

Each session will consist of 8 different training movements.

Each movement will be held for 6 seconds, followed by a 6-second rest.

Training will last for a total of 20 minutes per session.

Each movement will be performed in one set of 10 repetitions.

Frequency: 3 sessions per week for 6 weeks (18 sessions in total).

Table 1. core stability exercise

| **Week 1-2** | **Week 3-4** | **Week 5-6** |
| --- | --- | --- |
| Supine Transverse Abdominis Contraction Training: Move the belly button towards the spine. | Supine Transverse Abdominis Contraction with Opposite Arm and Leg Lift (Not Touching the Ground). | Supine Transverse Abdominis Contraction with Double Leg Bridge. |
| Supine Transverse Abdominis Contraction with Heel Slide. | Supine Transverse Abdominis Contraction with Double Leg Bridge. | Supine Transverse Abdominis Contraction with Air Cycling Movement. |
| Supine Transverse Abdominis Contraction with Lower Limb Lift. | Supine Transverse Abdominis Contraction with Single Leg Bridge (Left Leg). | Supine Transverse Abdominis Contraction with Crunch (Hands Touching Knees). |
| Supine Transverse Abdominis Contraction with Contralateral Limb Elevation. | Supine Transverse Abdominis Contraction with Single Bridge (Right Leg). | Kneeling Hand-Knee Position with Transverse Abdominis Contraction While Raising the Opposite Upper and Lower Limbs. |
| Supine Transverse Abdominis Contraction with Double Leg Bridge. | Kneeling side bridge (left side). | Kneeling side bridge with knee extended (left side). |
| Kneeling Hand-Knee Position with Transverse Abdominis Contraction While Raising the Upper Limb. | Kneeling side bridge (right side). | Kneeling side bridge with knee extended (right side). |
| Kneeling Hand-Knee Position with Transverse Abdominis Contraction While Raising the Lower Limb. | Prone position transverse abdominal contraction with opposite limb raise. | Prone position transverse abdominal contraction with opposite limb raise. |
| Kneeling Hand-Knee Position with Transverse Abdominis Contraction While Simultaneously Raising the Contralateral Upper and Lower Limbs. | Kneeling Hand-Knee Position with Transverse Abdominis Contraction While Simultaneously Raising the Contralateral Upper and Lower Limbs. | Plank Exercise |

#### ****Neuromuscular Electrical Stimulation (NMES) Treatment****

NMES will be applied to the bilateral multifidus and transversus abdominis/internal oblique muscles using an EN-Stim 4 neuromuscular stimulator (ENRAF-NONIUS B.V., Brusse, Netherlands).

****Electrode Placement:****

**Abdominal Electrodes:** One electrode will be placed 1 cm above the iliac crest along the mid-axillary line.The second electrode will be positioned 2 cm above and medial to the anterior superior iliac spine.**Lumbar Electrodes:** Positioned approximately 2 cm lateral to the L4 and L5 spinous processes.**Specifications:** Electrodes will measure 5 cm × 5 cm with hydrogel surfaces.

****Stimulation Parameters:****Pulse width: 200 μs;Frequency: 50 Hz;Contraction-rest cycle: Ramp-up: 1 second;Contraction: 4 seconds;Ramp-down: 1 second;Rest: 6 seconds.Total session duration: 20 minutes.Current intensity: Adjusted to elicit maximal muscle contraction without discomfort (e.g., burning sensation or severe tonic pain).

****Integration with Core Training:****

Core exercises will be performed during contraction phases, with rest during relaxation phases.Frequency: 3 sessions per week for 6 weeks (18 sessions in total).

#### ****Sham NMES Treatment****

- The current intensity will be set to the lowest level, insufficient to induce muscle contractions.
- Patients will not perceive contractions, but therapists will guide movement initiation and cessation by observing device indicators.
- Frequency: 3 sessions per week for 6 weeks (18 sessions in total).

### **Observational Indicators**

This study involves clinical routine evaluation methods, as detailed below:

#### ****Primary Outcome Measure****

**Surface Electromyography (sEMG) Assessment:**
Using the MegaWin ME6000-T8 surface electromyography system (Mega Electronics, Finland), sEMG assessments will be conducted before and after 6 weeks of treatment. The evaluations will monitor changes in the activation level and onset time of core muscles during lifting tasks.

#### ****Secondary Outcome Measures****

Assessments will be performed at baseline and after 6 weeks of treatment, including:

**Musculoskeletal Ultrasound:**The SONIMAGE HS1 ultrasound system (KONICA MINOLTA, Shanghai, China) will measure the thickness of the multifidus and abdominal muscles, as well as the cross-sectional area of the multifidus.

**Proprioception Testing:**Local sensory testing will assess proprioceptive improvements in the lumbar region.

**Oswestry Disability Index (ODI):**The ODI questionnaire will evaluate lumbar functional status.

**Visual Analogue Scale (VAS):**The VAS scale will assess pain intensity.

**Follow-Up Evaluation:**
Follow-up assessments will be conducted 6 months after treatment to evaluate the long-term effects of the intervention.

****Statistical Analysis****

Data analysis will be performed using SPSS 24.0 for Windows. Descriptive statistics for continuous variables will be presented as mean ± standard deviation. One-way ANOVA or Chi-square tests will be used to assess the differences in baseline characteristics across the three groups. A two-way ANOVA will be applied to compare the evaluation indicators between groups. Pearson's correlation analysis will be used to assess the relationship between changes in core muscle activation delay and relevant evaluation results. All statistical tests will be two-tailed, and a p-value of <0.05 will be considered statistically significant.

****Sample Size Calculation****

The sample size for this study is determined based on previous findings reported by Songjaroen et al. The calculation is based on the anticipated effect size for LM activation improvement, with a large effect size (Cohen's dz = 0.7), a statistical power of 80%, a significance level of 5%, and a standard deviation of 0.05 points. Using G*Power 3.1 software for the analysis, it is calculated that at least 43 participants are required. Considering an anticipated dropout rate of 20%, a total of 52 participants will be recruited for the study (26 in the experimental group and 26 in the control group).

****Data Management and Confidentiality****

All information related to the identity of participants will be kept strictly confidential. Relevant materials will not be disclosed to external parties beyond the scope permitted by applicable laws and/or regulations.

****Informed Consent****

Before being enrolled in the study, each participant will receive a complete and comprehensive explanation of the study's objectives, nature, procedures, potential benefits, and risks in written form. This will be provided by the investigator responsible for obtaining informed consent. Participants will be informed of their right to withdraw from the study at any time. Prior to enrollment, participants will be given adequate time to consider their decision. Only those who voluntarily agree to participate and sign the informed consent form will be included in the study.

****Adverse Events and Related Management Measures****

The neuromuscular electrical stimulation (NMES) therapy used in this study is a type of low-frequency electrotherapy. During the application of electrical stimulation, researchers will strictly follow the operating requirements specified in the device manual, including the placement of electrode pads and adjustment of stimulation parameters. There is a minimal risk of adverse events, such as minor electrical burns, in a very small number of participants. If an electrical burn occurs, the research team will provide appropriate care, including wound cleaning, sterilization with iodine-based solutions, and dressing with sterile gauze.

The core training program employed in this study is a standard exercise regimen commonly used for patients with chronic non-specific low back pain. Researchers will adhere to prescribed exercise protocols and ensure a safe training environment, including fall-prevention education. While the risk is minimal, some participants may experience mild adverse events, such as delayed onset muscle soreness (DOMS). If this occurs, the research team will provide supportive care, including massage therapy and heat applications, to alleviate the soreness.
