# Supplementary material for: Efficacy of superimposing neuromuscular electrical stimulation onto core stability exercise in patients with nonspecific low back pain: A study protocol for a randomized controlled trial
Source: PLoS One. 2025 May 7;20(5):e0322398. doi: 10.1371/journal.pone.0322398 (PMC12057853; doi:10.1371/journal.pone.0322398)
Supplement: S4 File — (DOCX) [file pone.0322398.s004.docx]

****Informed Consent Form**
**Signature Page****

I have read this informed consent form.
I have had the opportunity to ask questions, and all my questions have been answered.
I understand that participation in this study is voluntary.
I may choose not to participate in this study, or I may withdraw at any time by notifying the researcher without fear of discrimination or retaliation. My medical treatment and rights will not be affected in any way.
If I require other treatments, do not comply with the study protocol, or if an injury related to the study occurs, or for any other reason, the study physician may terminate my participation in this study.
I will receive a signed copy of this informed consent form.

**Participant Information**
Name (printed): ___________________
Signature: _______________________
Contact Information: ________________
Date: _______ Year _______ Month _______ Day

**For Participants Unable to Read or Sign Due to Lack of Capacity or Minor Status**
The guardian will act on behalf of the participant during the informed consent process and sign on their behalf.

**Guardian Information**
Name (printed): ___________________
Signature: _______________________
Relationship to Participant: ___________
Contact Information: ________________
Date: _______ Year _______ Month _______ Day

**For Illiterate Participants Unable to Read the Informed Consent Form**
A witness will observe the informed consent process and sign as a witness.

**Witness Information**
Name (printed): ___________________
Signature: _______________________
Contact Information: ________________
Date: _______ Year _______ Month _______ Day

**Researcher Statement**
I have accurately explained this document to the participant, who has read and understood it. I confirm that the participant has had the opportunity to ask questions and has voluntarily agreed to participate.

**Researcher Information**
Name (printed): ___________________
Signature: _______________________
Contact Information: ________________
Date: _______ Year _______ Month _______ Day
