# Supplementary material for: Efficacy of superimposing neuromuscular electrical stimulation onto core stability exercise in patients with nonspecific low back pain: A study protocol for a randomized controlled trial
Source: PLoS One. 2025 May 7;20(5):e0322398. doi: 10.1371/journal.pone.0322398 (PMC12057853; doi:10.1371/journal.pone.0322398)
Supplement: Attachment — Submitted filename: Reviewers comment.docx [file pone.0322398.s005.docx]

The aim of this study is to assess the efficacy of superimposing neuromuscular electrical stimulation (NMES) onto Core Stability Exercise (CSE) for improving muscle activation, function, and proprioception as well as reducing pain in non-specific low back pain (NSLBP) patients.

This study is very clear and detailed regarding scientific content, experimental protocol, and physiological and clinical measurements. I believe the authors have devised a protocol that will provide significant details regarding NSLBP treatment through the CSE and NMES use. The results of this study can improve existing rehabilitation protocols to manage chronic pain in these patients.

I have only some questions and suggestions to make to the authors, to improve their study protocol.

Pag. 4, line 153 ‘Eligibility criteria’. Were these inclusion/exclusion criteria chosen based on previous studies? If so, which ones?

Pag. 5, line 208 ‘Table 1. core stability exercise’: The exercises and their progress over the weeks are clearly shown in this table. It would be helpful if the authors specified that the progression of exercises is tailored to the individual patient's abilities. As an example, it is not stated that a patient at week 3 is already capable of doing these exercises because he may be suffering from lumbar pain. In other words, the general progression is fine, but the timing of it should depend on the individual characteristics. I suggest the authors to specify this point.

Pag. 7, line 217 ‘The stimulation pulse width will be set to 200 μs,..’: Why did the authors choose this pulse width? Could they mention the source from which they took this parameter?

Pag. 7, lines 225 – 227 ‘Each session will consist of 8 different exercises, performing 1 set of 10 repetitions per exercise, lasting 20 minutes per session, once per day, 3 days per week.’: Also here, why have they chosen this protocol? Is there a progression in the number of series/repetitions, or an increase in the intensity of NMES in the experimental group, during the 6 weeks of intervention? After a few weeks you may need to increase the load of the training session (always respecting the patient’s pain).
