# Supplementary material for: Efficacy of superimposing neuromuscular electrical stimulation onto core stability exercise in patients with nonspecific low back pain: A study protocol for a randomized controlled trial
Source: PLoS One. 2025 May 7;20(5):e0322398. doi: 10.1371/journal.pone.0322398 (PMC12057853; doi:10.1371/journal.pone.0322398)
Supplement: Attachment — Submitted filename: Response to Reviewers.docx [file pone.0322398.s007.docx]

**Revision Notes**

Submission ID: PONE-D-24-54076

“Efficacy of Superimposing Neuromuscular Electrical Stimulation onto Core Stability Exercise in Patients with Nonspecific Low Back Pain: A Study Protocol for a Randomized Controlled Trial”

We thank the editors for allowing us to revise our paper. Here below is our description on revision to the reviewers’ comments and our responses are in italics.

****Reviewer #1:**** This is a well designed protocol for a randomised controlled trial to study the efficacy of superimposing NMES onto core stability exercise in patients with nonspecific low back pain. I have few comments.

1. The test statistic used to calculate sample size needs to be mentioned.

**** Thank you for pointing this out. The test statistic used to calculate the sample size was based on a mixed ANOVA (Fixed effects, main effects and interactions). This was selected to detect a statistically significant difference in the primary outcome measure, the improvement in LM activation, between the intervention group and the control group. The calculation assumed a significance level (α) of 0.05, a power (1-β) of 0.8, and an effect size derived from previous studies examining similar interventions for NSLBP. We have now updated the manuscript to include this information for clarity.*

2. If the participants cannot be blinded then what is the point of using sham-NMES?

****Thank you for raising this important question. While we acknowledge that participant blinding is challenging in this study due to the physical sensation elicited by NMES, the use of sham-NMES serves a critical purpose in minimizing performance bias. Sham-NMES provides a control condition that mimics the setup and experience of the intervention without delivering therapeutic electrical stimulation. This approach allows us to isolate the effects of the active NMES on clinical outcomes (e.g., pain, proprioception, muscle activation) by ensuring that any observed improvements are not simply due to placebo effects, psychological factors, or the participants' awareness of receiving NMES treatment. Additionally, sham-NMES contributes to maintaining blinding for outcome assessors and data analysts, who remain unaware of the participants' group allocation. This further enhances the methodological rigor and validity of the study. We have clarified this rationale in the manuscript to address your concern and provide better justification for the use of sham-NMES. Thank you for highlighting this point.*

3. Why 6-weeks intervention period instead of 8-weeks?

****Thank you for your insightful question regarding the duration of the intervention. We chose a 6-week intervention period based on several factors. Firstly, previous research indicates that significant improvements in pain, muscle function, and proprioception in NSLBP patients can often be observed within a 6-week period. This timeframe has been shown to be sufficient for neurophysiological changes and muscle activation adaptations, especially in CSE and NMES interventions[1]. Moreover, a 6-week duration strikes a balance between providing adequate time for measurable physiological adaptations while ensuring feasibility for participants, minimizing drop-out rates, and maintaining participant engagement. That said, we are open to exploring the benefits of extending the intervention period in future studies, as longer durations may yield additional insights into the long-term effects of combined CSE and NMES.*

[1] *Gomes-Neto M, Lopes JM, Conceicao CS, et al. Stabilization exercise compared to general exercises or manual therapy for the management of low back pain: A systematic review and meta-analysis. Phys Ther Sport. 2017, 23:136-142. doi: 10.1016/j.ptsp.2016.08.004.*

4. Discussion section seems like repetition of the introduction. I think, the discussion section should summarise the protocol and justify the methods adopted.

****Thank you for your observation regarding the Discussion section. We recognize that parts of the current Discussion section may overlap with the Introduction, which could reduce its focus and clarity. To address your concern, we will revise the discussion section to better summarize the protocol and justify the methods adopted, rather than repeating background information. Specifically, the revised section will include:*

1. *A concise summary of the study design, including the rationale for using a combination of CSE and NMES.*
2. *A clear explanation of how the study addresses gaps in previous research by focusing on proprioception and both structural and functional changes in the lumbar musculature in NSLBP patients.*
3. *A justification for the chosen outcome measures (e.g., surface EMG, real-time ultrasound imaging, proprioceptive tests) and their relevance to NSLBP mechanisms.*
4. *An explanation of why the 6-week intervention period and specific NMES-CSE combination protocol were selected, supported by evidence from prior studies.*

*We will ensure that the revised section focuses on interpreting and justifying the protocol rather than repeating the broader background provided in the Introduction. We appreciate your valuable feedback and will implement these changes to strengthen the clarity and coherence of the discussion section.*

****Reviewer #2:**** The aim of this study is to assess the efficacy of superimposing neuromuscular electrical stimulation (NMES) onto Core Stability Exercise (CSE) for improving muscle activation, function, and proprioception as well as reducing pain in non-specific low back pain (NSLBP) patients.
This study is very clear and detailed regarding scientific content, experimental protocol, and physiological and clinical measurements. I believe the authors have devised a protocol that will provide significant details regarding NSLBP treatment through the CSE and NMES use. The results of this study can improve existing rehabilitation protocols to manage chronic pain in these patients.
I have only some questions and suggestions to make to the authors, to improve their study protocol.

Pag. 4, line 153 ‘Eligibility criteria’. Were these inclusion/exclusion criteria chosen based on previous studies? If so, which ones?

****Thank you for your constructive feedback on our manuscript. The inclusion and exclusion criteria were indeed based on findings from previous studies, which provided a foundation for ensuring the recruitment of an appropriate and homogenous sample of NSLBP patients for this study. This ensures transparency and demonstrates that the criteria were evidence-based, aligning with current clinical practice and research in NSLBP. Specifically:*

1. *Patients diagnosed with NSLBP lasting more than 3 months: Chronic NSLBP is defined as pain persisting for more than 12 weeks without a specific identifiable cause[1-2]. This time frame is widely used in studies as it differentiates chronic from acute or subacute conditions.*
2. *Age range of 18–65 years: This range encompasses adults who are generally active and capable of participating in CSE and NMES. It is consistent with other NSLBP studies[3].*
3. *Specific spinal pathologies or red flags (e.g., fractures, tumors, inflammatory conditions): Red flag conditions can mimic NSLBP but require different treatments. Exclusion is standard practice in NSLBP trials [4].*
4. *Pregnancy: Hormonal and biomechanical changes during pregnancy can influence lumbar biomechanics and pain mechanisms, potentially confounding results [5].*
5. *Previous lumbar surgery or ongoing invasive treatments: Structural changes or ongoing treatments can affect muscle activation patterns and proprioception, which would interfere with the evaluation of NMES and CSE effects.*
6. *Neurological or musculoskeletal conditions affecting movement or balance: Conditions such as stroke or severe osteoarthritis alter motor control strategies and muscle recruitment patterns, which would confound the intervention’s effects.*
[1] *Airaksinen O, Brox JI, Cedraschi C, et al. European Guidelines for the Management of Chronic Nonspecific Low Back Pain. Eur Spine J. 2006;15(S2):S192-S300. doi: 10.1007/s00586-006-1072-1.*
[2] *Ma K, Zhuang ZG, Wang L, et al. The Chinese Association for the Study of Pain (CASP): Consensus on the Assessment and Management of Chronic Nonspecific Low Back Pain. Pain Res Manag. 2019, 2019:8957847. doi: 10.1155/2019/8957847.*
[3] *Gomes-Neto M, Lopes JM, Conceição CS, et al. Stabilization Exercise Compared to General Exercises or Manual Therapy for the Management of Low Back Pain: A Systematic Review and Meta-Analysis. Phys Ther Sport. 2017;23:136-142. doi: 10.1016/j.ptsp.2016.08.004.*
[4] *Maher C, Underwood M, Buchbinder R. Non-Specific Low Back Pain. Lancet. 2017;389(10070):736-747. doi: 10.1016/S0140-6736(16)30970-9.*
[5] *Vleeming A, Albert HB, Ostgaard HC, et al. European Guidelines on the Diagnosis and Treatment of Pelvic Girdle Pain. Eur Spine J. 2008;17(6):794-819.doi: 10.1007/s00586-008-0602-4.*

    Pag. 5, line 208 ‘Table 1. core stability exercise’: The exercises and their progress over the weeks are clearly shown in this table. It would be helpful if the authors specified that the progression of exercises is tailored to the individual patient's abilities. As an example, it is not stated that a patient at week 3 is already capable of doing these exercises because he may be suffering from lumbar pain. In other words, the general progression is fine, but the timing of it should depend on the individual characteristics. I suggest the authors to specify this point.

****We appreciate the reviewer’s observation regarding the progression of CSE and agree that individualizing the timing of progression based on the patient’s abilities and condition is essential. To address this concern, we have added clarification to the manuscript as follows: The progression of CSE detailed in Table 1 represents a general framework for advancing exercise difficulty over the 6-week intervention period. However, we acknowledge that not all patients may progress at the same rate due to variability in pain levels, functional capacity, or motor control impairments. To accommodate individual differences, the exercise progression will be tailored to each patient’s abilities and clinical presentation. The supervising therapist will evaluate the patient’s pain, functional status, and motor performance at each session. Progression to subsequent levels will only occur when the patient demonstrates: Adequate pain tolerance during the current exercise level (i.e., no significant exacerbation of symptoms). Sufficient motor control and stability to safely perform the next level of exercises without compensatory movement patterns.*

*This individualized approach ensures that patients who may be experiencing higher levels of lumbar pain or functional limitations can proceed at a pace suited to their capabilities, minimizing the risk of aggravating symptoms while promoting optimal therapeutic outcomes. This modification ensures the flexibility and practicality of the protocol while maintaining its adherence to evidence-based practice. Thank you for this valuable suggestion.*

Pag. 7, line 217 ‘The stimulation pulse width will be set to 200 μs,..’: Why did the authors choose this pulse width? Could they mention the source from which they took this parameter?

****We thank the reviewer for raising this important question regarding the rationale for selecting a pulse width of 200 μs for NMES. The stimulation pulse width will be set to 200 μs to ensure effective activation of the targeted motor units in the deep lumbar muscles, such as the lumbar multifidus (LM) and transverse abdominis (TrA). This pulse width was chosen based on prior studies that have demonstrated its efficacy in selectively recruiting motor units within the deep stabilizing musculature, while minimizing patient discomfort and avoiding activation of superficial muscles. Specifically, Baek et al. (2014) utilized a pulse width of 200 μs to successfully activate the deep lumbar muscles with minimal discomfort in NMES interventions targeting the LM[1]. Similarly, Maffiuletti (2010) highlighted that pulse widths between 200–300 μs are optimal for achieving efficient motor unit recruitment in neuromuscular stimulation protocols[2]. The choice of 200 μs thus balances the effectiveness of motor unit recruitment and patient comfort during the intervention.We have added relevant explanations in the discussion section. Thank you again for your valuable suggestions.*

[1] *Baek SO, Ahn SH, Jones R, et al. Activations of deep lumbar stabilizing muscles by transcutaneous neuromuscular electrical stimulation of lumbar paraspinal regions. Ann Rehabil Med. 2014;38(4):506-513. doi:10.5535/arm.2014.38.4.506.*
[2] *Maffiuletti NA. Physiological and methodological considerations for the use of neuromuscular electrical stimulation. Eur J Appl Physiol. 2010;110(2):223-234. doi:10.1007/s00421-010-1502-y.*

Pag. 7, lines 225 – 227 ‘Each session will consist of 8 different exercises, performing 1 set of 10 repetitions per exercise, lasting 20 minutes per session, once per day, 3 days per week.’: Also here, why have they chosen this protocol? Is there a progression in the number of series/repetitions, or an increase in the intensity of NMES in the experimental group, during the 6 weeks of intervention? After a few weeks you may need to increase the load of the training session (always respecting the patient’s pain).

****Thank you for pointing out the need for further clarification regarding the exercise protocol and progression in the intervention plan. The protocol of "8 different exercises, performing 1 set of 10 repetitions per exercise, lasting 20 minutes per session, once per day, 3 days per week" was designed based on existing evidence from studies that demonstrated effective outcomes using similar schedules. For example, Baek et al. (2014) explored NMES application in lumbar stabilizing muscles[1], and Maffiuletti (2010) provided physiological insights that guided the session duration and frequency to optimize neuromuscular benefits without inducing fatigue[2]. Progression is indeed incorporated into the intervention plan to ensure continuous adaptation and engagement of the participants' neuromuscular systems. Specifically: The exercises outlined in Table 1 are progressively tailored based on individual participant abilities and pain levels. While all participants start with simpler movements in the initial weeks, the complexity and demands of exercises increase over time. For example, CSE evolve from static stabilization tasks to dynamic, multi-joint tasks involving greater motor control and strength requirements. In the experimental group, NMES intensity is adjusted weekly based on participant feedback and tolerability, as well as to match improvements in muscle strength and activation capacity. Intensity increases are guided by the participant’s perception of a "strong but comfortable" stimulation level and monitored through visible muscle contractions. This progressive design aims to prevent overloading participants early in the program while ensuring gradual and safe improvements in muscle activation, strength, and control. Additionally, respecting the patient's pain levels is a priority, and adjustments are made as necessary to accommodate individual needs and avoid exacerbating symptoms. We have made the relevant modifications in the methods section of the article. Thank you again for your valuable suggestions.*

[1] *Baek SO, Ahn SH, Jones R, et al. Activations of deep lumbar stabilizing muscles by transcutaneous neuromuscular electrical stimulation of lumbar paraspinal regions. Ann Rehabil Med. 2014;38(4):506-513.*
[2] *Maffiuletti NA. Physiological and methodological considerations for the use of neuromuscular electrical stimulation. Eur J Appl Physiol. 2010;110(2):223-234.*

****Reviewer #3:**** The proposed research outlines a clinical randomized controlled trial investigating the effects of a six-week training program involving neuromuscular electrical stimulation (NMES) superimposed on core stability exercises (CSE) in individuals with non-specific low back pain (NSLBP). The protocol aims to assess physiological, functional, and proprioceptive outcomes following the exercise protocol compared to a control group performing only sham stimulation. Overall the study is well written, and the methodology is faitly robus. However, there are several issues in the elaboration of the study's rationale and other minor shortcomings that need to be addressed.

Major issues

- Although the introduction section includes a well-written section discussing the effects of superimposed NMES on spinal and supraspinal mechanisms, it lacks sufficient detail regarding the physiological, functional, and proprioceptive aspects that are later outlined as assessment measures in the methods section. The study's rationale would significantly benefit if these aspects were clearly outlined in the introduction.

****We acknowledge that the introduction would benefit from a more thorough discussion of the physiological, functional, and proprioceptive components that are key assessment measures in the study. In response to your feedback, we will revise the introduction section. The specific modifications will be marked in red. By incorporating these physiological, functional, and proprioceptive aspects into the introduction, we aim to provide a clearer rationale for the study's assessment measures, and help readers understand the mechanisms through which the NMES superimposed on CSE is expected to produce clinical benefits for NSLBP patients. This revision will also align the rationale with the methodology and the specific outcomes being assessed, creating a cohesive narrative that justifies the proposed approach. Thank you again for your suggestion, it will greatly improve the overall clarity and justification of the study.*

- The methods also lacks comprehensive information on the electrical stimulation characteristics such as NMES frequency, waveform, sensitivity of the NMES modulation.

****This revised Methods section now includes detailed descriptions of the NMES parameters such as frequency, waveform, pulse width, intensity, duty cycle, and modulation, which are critical for replicating the stimulation protocol and understanding the rationale behind its design. Thank you for your valuable suggestions.*

- The duration of the exercise program and the overall protocol lack sufficient references to justify the choices made by the authors. Please include appropriate citations to support the overall CSE protocol.

****The duration and structure of the CSE program were designed based on evidence from previous studies indicating the efficacy of similar protocols in patients with NSLBP. Research suggests that exercise interventions lasting 6 to 8 weeks are optimal for improving both pain and function in NSLBP patients[1-2]. A 6-week period is commonly recommended for intervention protocols, as it provides sufficient time for neuromuscular adaptation and improved motor control without overwhelming patients[3]. This duration has been shown to result in clinically significant improvements in pain, function, and muscle strength[4]. Moreover, NMES superimposed on exercise training has demonstrated enhanced outcomes, particularly in motor control and muscle activation in individuals with chronic low back pain[5]. The relevant content and references have been added to the methods section of the article. Thank you for your valuable suggestions.*

[1] *Niederer, D., & Mueller, J. (2020). Sustainability effects of motor control stabilisation exercises on pain and function in chronic nonspecific low back pain patients: A systematic review with meta-analysis and meta-regression. PLoS One, 15(12), e0227423.*
[2] *Shanbehzadeh, S., ShahAli, S., Hides, J., et al. (2022). Effect of Motor Control Training on Trunk Muscle Morphometry, Pain, and Disability in People With Chronic Low Back Pain: A Systematic Review and Meta-Analysis. Journal of Manipulative and Physiological Therapeutics, 45(3), 202-215.*
[3] *Bagheri, R., Parhampour, B., Pourahmadi, M., et al. (2019). The Effect of Core Stabilization Exercises on Trunk-Pelvis Three-Dimensional Kinematics During Gait in Non-Specific Chronic Low Back Pain. Spine (Phila Pa 1976), 44(14), 927-936.*
[4] *Hwang, U. J., Jung, S. H., Kim, H. A., et al. (2020). Effect of Abdominal Electrical Muscle Stimulation Training With and Without Superimposed Voluntary Muscular Contraction on Lumbopelvic Control. Journal of Sport Rehabilitation, 29(6), 1137-1144.*
[5] *Weissenfels, A., Wirtz, N., Dormann, U., et al. (2019). Comparison of Whole-Body Electromyostimulation versus Recognized Back-Strengthening Exercise Training on Chronic Nonspecific Low Back Pain: A Randomized Controlled Study. Biomedical Research International, 2019, 5745409.*

Minor issues

Introduction

-L 95/L 98. "combined with", "overlaying". For better clarity please use "superimposed on" throughout the entire document.

****I have replaced "combined with" with "superimposed on" for better clarity in accordance with your request.*

Methods

- L 130. Is there any reference that confirms that 6 weeks of CSE are enough to carry significant physiological/functional adaptation?

****Yes, there are several studies that suggest that 6 weeks of CSE can lead to significant physiological and functional adaptations[1-2]. While the exact duration can vary based on individual circumstances, 6 weeks is often a commonly used timeframe for observing noticeable changes in muscle strength, proprioception, and pain reduction in the context of low back pain. These studies suggest that a 6-week duration for CSE is both sufficient and effective for achieving meaningful functional and physiological improvements in patients with low back pain. Thank you for your valuable suggestions.*

[1] *Shanbehzadeh S, ShahAli S, Hides J, et al. Effect of motor control training on trunk muscle morphometry, pain, and disability in people with chronic low back pain: A systematic review and meta-analysis. J Manipulative Physiol Ther. 2022, 45:202-215.*
[2] *Niederer D, Mueller J. Sustainability effects of motor control stabilisation exercises on pain and function in chronic nonspecific low back pain patients: A systematic review with meta-analysis and meta-regression. PLoS One. 2020, 15:e0227423.*

- L156. The phrase "no treatment intervention utilized" is unclear. Please rephrase for better clarity. What exactly does this refer to?.

****Thank you for your valuable suggestions. We have replaced the original unclear expressions with "No received CSE or NMSE therapy within the past month.".*

-L 192. Specify what is meant by "necessary adjustments to the treatment protocol." What adjustments are planned, and under what circumstances will they be made?

****Thank you for your detailed and helpful feedback regarding the methods of our*

*Study. Here’s a revised version of the content, incorporating more detailed explanations of the strategies and adjustments that will be made: Assessors will maintain regular communication with participants throughout the study to monitor their progress and address any discomfort or challenges. Participants will be encouraged to promptly report any increase in pain, discomfort, or difficulty in performing exercises or tolerating NMES. If any issues arise, the research team will assess the individual’s condition and make necessary adjustments to the treatment protocol. These adjustments may include modifying the intensity or duration of NMES, altering the difficulty level of exercises, or providing alternative movements to ensure proper execution while minimizing pain. If NMES settings prove intolerable, parameters such as frequency, intensity, or pulse duration may be adjusted to improve comfort without compromising effectiveness. Additionally, the progression of CSE will be personalized, allowing flexibility in exercise advancement based on each participant’s physical capability. To enhance adherence, assessors will offer continuous guidance, encouragement, and regular check-ins, reinforcing motivation and addressing concerns in real time. By implementing these strategies, the study aims to optimize participant engagement, ensure a tolerable and effective intervention, and maximize the benefits of the treatment protocol.*

-L 213. Please clarify which electrode will function as the cathode and which as the anode in this NMES configuration.

****Thank you for your detailed and helpful feedback regarding the methods of our Study. Here’s a revised version of the content: For the NMES configuration, the abdominal electrodes will be positioned as follows: the cathode (negative electrode) will be placed 1 cm above the iliac crest along the mid-axillary line, while the anode (positive electrode) will be located 2 cm medial to the anterior superior iliac spine. For the lumbar electrodes, the cathode (negative electrode) will be positioned approximately 2 cm lateral to the spinous processes of L4 and L5, with the anode (positive electrode) placed at a distance from the cathode to ensure optimal current flow through the lumbar region.*

-L 217. In line with the comment above. Which NMES pulse frequency will be used? Based on what rationale?

****Thank you for your valuable suggestions. The stimulation frequency will be set at 50 Hz, a commonly used frequency for muscle strengthening. This frequency has been shown to effectively recruit motor units while minimizing discomfort, promoting optimal muscle contraction for rehabilitation purposes[1-2].*

[1] *Hicks GE, Sions JM, Velasco TO, et al. Trunk muscle training augmented with neuromuscular electrical stimulation appears to improve function in older adults with chronic low back pain: A randomized preliminary trial. Clin J Pain. 2016;32(10):898-906. doi: 10.1097/AJP.0000000000000335.*
[2] *Bilgin S, Temucin CM, Nurlu G, et al. Changes in deep lumbar stabilizing muscle thickness by transcutaneous neuromuscular electrical stimulation in patients with low back pain. J Back Musculoskelet Rehabil. 2013;26(3):261-266. doi: 10.3233/BMR-130385.*

-L 225-227. Include appropriate references to justify the selection of the exercise protocol.

****Thank you for your valuable suggestions. The duration and structure of the CSE program were designed based on evidence from previous studies indicating the efficacy of similar protocols in patients with NSLBP[1-3].*

[1] *Niederer, D., & Mueller, J. (2020). Sustainability effects of motor control stabilisation exercises on pain and function in chronic nonspecific low back pain patients: A systematic review with meta-analysis and meta-regression. PLoS One, 15(12), e0227423.*
[2] *Hwang, U. J., Jung, S. H., Kim, H. A., et al. (2020). Effect of Abdominal Electrical Muscle Stimulation Training With and Without Superimposed Voluntary Muscular Contraction on Lumbopelvic Control. Journal of Sport Rehabilitation, 29(6), 1137-1144.*
[3] *Weissenfels, A., Wirtz, N., Dormann, U., et al. (2019). Comparison of Whole-Body Electromyostimulation versus Recognized Back-Strengthening Exercise Training on Chronic Nonspecific Low Back Pain: A Randomized Controlled Study. Biomedical Research International, 2019, 5745409.*

-L244-245. Based on what evidence did the authors choose a interelectrode distance of 2.5 cm?

****Thank you for your constructive feedback on our manuscript. Previous research articles suggest that a distance of 2.5 cm between electrodes is commonly used in the lifting process, as they provide a good balance between comfort and effectiveness without causing excessive current density at the skin surface [1].*

*[1]Suehiro T, Ishida H, Kobara K, et al. Altered trunk muscle recruitment patterns during lifting in individuals in remission from recurrent low back pain. J Electromyogr Kinesiol. 2018 Apr;39:128-133. doi: 10.1016/j.jelekin.2018.02.008.*

-L 265-269. Regarding EMG processing, the procedures for manually selecting activation timings need further clarification. For instance: Is there a specific signal threshold used? How are "baseline levels" defined? Provide clear, step-by-step details.

****Thank you for your detailed and helpful feedback regarding the methods of our Study. For EMG processing, the procedures for manually selecting activation timings are as follows: Raw EMG signals will first be filtered using a band-pass filter (20–450 Hz) to remove noise and artifacts. The baseline EMG levels will be defined by calculating the root mean square (RMS) value of a 500 ms resting period before the onset of muscle activation. Muscle activation will be identified when the EMG signal exceeds 3 standard deviations (SD) above the baseline RMS for a minimum duration of 50 ms. If necessary, trained assessors will visually inspect and manually adjust the activation timing to account for signal variations and artifacts, ensuring consistency across trials. The above content has been added to the Primary Outcome Measures section of the article.*

-L 314. Add a space after "..located at S1."

****Thank you very much for your reminder and the corrections made in the text.*

-L 347. If a statistical design comparing groups (between-group) and repeated measures over time (within-group) is used, define it as a mixed ANOVA.

****Thank you very much for your valuable suggestions. I have made the necessary revisions in the text.*

Discussion

While the discussion of the RCT is well-written overall, it lacks clarity regarding the EMG-related improvements anticipated from the proposed exercise protocol. Please elaborate on how the protocol is expected to influence EMG outcomes and the significance of these improvements.

****Thank you for your detailed and helpful feedback regarding the discussion of our Study. Here’s a revised version of the content: This study employs sEMG technology to assess the activation patterns of deep core muscles such as the LM and TrA. Its high sampling rate and sensitivity help capture dynamic muscle changes, particularly in quantifying muscle activation timing differences, which further reveal motor control impairments in patients[1][2]. The anticipated improvements in sEMG outcomes include increased muscle activation, with NMES enhancing motor unit recruitment, leading to more consistent activation of the TrA and LM during movement. This is particularly important for individuals with NSLBP, as these muscles often fail to activate effectively during functional tasks. The protocol also aims to improve motor control through neuromuscular system retraining, enhancing proprioceptive feedback and muscle coordination. This will result in better lumbar stability during movement, reducing the risk of injury. Over time, improvements in these sEMG outcomes are expected to correlate with functional benefits, such as reduced pain, increased stability, and improved movement patterns, allowing individuals to perform daily activities with less discomfort and greater ease, potentially reducing pain recurrence.*

[1] *Suo, M., L. Zhou, J. Wang, H. Huang, J. Zhang, T. Sun, X. Liu, et al. "The Application of Surface Electromyography Technology in Evaluating Paraspinal Muscle Function." Diagnostics (Basel) 14, no. 11 (May 24 2024).*
[2] *McManus, L., G. De Vito, and M. M. Lowery. "Analysis and Biophysics of Surface Emg for Physiotherapists and Kinesiologists: Toward a Common Language with Rehabilitation Engineers." Front Neurol 11 (2020): 576729.*
